# Exploring healthcare professionals’ views and experiences of having access to a rehabilitation coordinator in their work in psychiatric care: a qualitative descriptive study

**DOI:** 10.1186/s12913-026-15919-8

**Published:** 2026-10-27

**Authors:** Åsa Andersén, Beatrice Carpentsier, Maria Carlsson, Erik Berglund, Ingrid Anderzén, Kjerstin Larsson

**Affiliations:** Department of Public Health and Caring Sciences, Uppsala University, Box 564, Uppsala, SE-751 22 Sweden

**Keywords:** Healthcare workers, Mental disorders, Sick leave, Coordination, Return-to-work, Vocational rehabilitation, Interviews

## Abstract

**Background:**

In Sweden, rehabilitation coordinators (RCs) have been introduced into healthcare service to offer coordination and support to patients, with the aim of streamlining sick leave and rehabilitation processes and promoting patients’ return-to-work (RTW)/entry into work. Before the introduction of RCs, there was no corresponding function with mainly this specific focus and responsibility. The present study explores healthcare professionals’ (HCPs’) views and experiences of having access to a RC within psychiatric care.

**Methods:**

This descriptive qualitative study involved individual semi-structured interviews with 12 HCPs in psychiatric care. Data were analysed using inductive thematic analysis.

**Results:**

Two main themes were identified: “A specific function that improves the ability to focus on and support vocational rehabilitation” and “Uncertainty and confusion regarding roles and contributions within vocational rehabilitation”. The introduction of a RC was viewed positively, enhancing the possibilities to focus on and offer patients coordination and vocational rehabilitation (VR) efforts. The HCPs felt they lacked both the time and knowledge to manage these tasks within their current work situation. By sharing knowledge, taking responsibility for, and coordinating the patients’ VR process, the RC was seen as a valuable resource that contributed to better working situation for HCPs. However, the introduction of the RC also brought about uncertainty and confusion, particularly linked to the RC function and the role of HCPs within VR.

**Conclusions:**

The RC’s focus and in-depth knowledge of VR addressed a gap within psychiatric healthcare units. This contributed to providing more extensive support in patients’ VR processes and improved the working environment for HCPs. To minimise inequalities in patient care and support the implementation of the RC function within psychiatric care, it is essential to clarify the roles and responsibilities of both RCs and HCPs in patients’ VR processes. The findings of this study may guide and support the implementation of RCs across various care units, potentially extending beyond psychiatric care.

**Trial registration:**

Clinicaltrials.gov Register Platform (ID NCT03729050 November 2018).

**Supplementary Information:**

The online version contains supplementary material available at https://doi.org/10.1186/s12913-026-15919-8.

## Background

An important change in the Swedish healthcare system is the introduction of rehabilitation coordinators (RCs). The need to develop the role of healthcare in managing the sick leave process, supporting the patient, and increasing collaboration between healthcare and different actors in the rehabilitation process contributed to the development of this new function [1, 2]. Initially, RCs were introduced within primary care. However, today RCs can also be found within specialist areas, such as the psychiatric care. In 2020, Swedish law established that the healthcare system must offer a coordination service for patients on sick leave, if needed, to support their return to work (RTW)/entry into work [3]. The essential tasks of RCs include managing coordination and collaboration within the healthcare system, facilitating external collaboration and providing support to patients in their rehabilitation process; however, their role does not include providing treatment. RCs can contribute with knowledge related to sick leave and the rehabilitation process within the healthcare system, as well as advise healthcare professionals (HCPs) [1].

Rehabilitation has different meanings in different contexts. In the present study, the term vocational rehabilitation (VR) is used for the rehabilitation process that the RCs are assigned to make more efficient in order to facilitate the goal of RTW/entry into work or studies. VR can include both medical and non-medical efforts and aims to support individuals with physical-, mental- or social disabilities in RTW/entry into work. This can include coordination, planning, assessments (e.g. functional- and work capacity) [4].

Healthcare services should, based on their competence, work to promote patients’ health and RTW/entry into work [5]. They have the medical responsibility for investigations, diagnostics, treatment and medical rehabilitation, as well as for issuing sick leave certificates [1]. Various professions can have different roles in supporting patients’ ability to work. Physicians are responsible for the medical care and rehabilitation of their patients, make referrals, and initiate investigations and treatments. If needed by the patient, other HCPs may also provide different social- and work-oriented support [1].

The healthcare service’s work with sick leave and rehabilitation is shared with other external actors and includes various efforts to promote work ability and thereby RTW/entry into work [6]. These actors partly have their own areas of responsibilities within the patient’s VR process but also share responsibilities. They can complement each other and help patients through different efforts to promote their possibility for RTW/entry into work. The Swedish Social Insurance Agency (SIA) is responsible for assessing the individual’s right to compensation from the social insurance in the event of reduced work ability, investigating the need for VR efforts, as well as coordinating them [6]. The employer, (if there is any) has the responsibility for the VR [5]. The Swedish Public Employment Service (PES) is responsible for the VR if there is no employer, and the municipality is responsible for the social rehabilitation [7]. These different actors can influence the VR process in various ways due to their diverse interests and perspectives [8]. However, a lack of collaboration among these involved actors can prolong patients’ sick leave duration [9]. A newly published review found that a lack of trust, negative attitudes, and differing goals and perspectives hinder engagement and collaboration within the mental healthcare sector [10]. To prevent the VR process from becoming confusing or uncertain, it is also important that all involved actors, including the patient, understand each other’s roles and responsibilities [8].

HCPs in mental healthcare have reported that patients often require support with RTW/entry into work, as employment, in any form, is seen to improve mental health and provide a sense of purpose and structure to everyday life [11]. Mental disorders can sometimes lead to feelings of being overwhelmed or hopelessness regarding one’s situation, which may result in difficulties for patients in initiating, handling and managing different contacts and responsibilities independently. Therefore, support from an RC, tailored to patients’ specific difficulties and needs, was deemed to promote the VR process, and motivating the patients to engage with it and reduce their stress [11]. The importance of coordination within this target group has also been emphasised in another study [12]. To facilitate the patient’s opportunity to RTW/entry into work, the implementation of RCs within healthcare services may be of importance.

A similar coordination role to that of RCs in the Swedish healthcare system also exists internationally, where it is often referred to as RTW coordinators (RTWCs), although they are often found in companies and insurance agencies [13]. Previous findings on the impact of RTWC efforts on RTW and sick leave are inconsistent. A meta-analysis reported positive effects on the proportion of individuals returning to work for patients with mental- or musculoskeletal disorder [14]. However, another review found that RTWC efforts delayed the time to RTW for individuals with mental disorders (e.g. anxiety and depression) or sub-clinical mental ill-health concerns [15]. A study by Skarpaas et al. revealed a delay in RTW and no difference in full RTW rates for employees with mental- or musculoskeletal disorder compared to those who did not receive RTWC assistance [16]. A recent evaluation conducted within the Swedish primary care [17] reported that efforts by a RC had positive effects on sick leave (i.e. lower rates, shorter duration and fewer repeated sick leaves) among patients with mental- or musculoskeletal disorders compared with the control group. However, there was no effect on RTW or the degree of sick leave for patients with a combination of these diagnoses [17]. The different results across various studies may depend on, e.g. study design, the length- and content of the RC intervention, length of sick leave, employment status and specific diagnoses.

There are also studies investigating the experiences of RC efforts from the perspective of patients. For example, studies conducted in the Swedish context have shown that RCs can be beneficial for patients [18–20]. Patients perceived RCs as helpful in initiating and promoting coordination within healthcare services, facilitating communication with employers and providing advice and information [18]. Furthermore, patients described that RCs contribute to coherent and continuous support, providing structure and clarity throughout the rehabilitation process [19].

Previous studies have mainly focused on perspectives regarding outcomes and experiences for patients in relation to RCs. Considering the different roles and responsibilities of HCPs in patient care, this study aims to explore HCPs’ views and experiences of having access to a rehabilitation coordinator in their work within psychiatric care, where the function is relatively new. This knowledge is still limited and needs to be developed to support the implementation of RCs.

## Methods

### Study design

The present study has a descriptive qualitative design to meet the aims of the study [21]. The reporting of the study follows the Consolidated criteria for reporting qualitative research (COREQ) checklist, ensuring quality by reporting important aspects of the research process [22].

### Primary trial

This study is part of a larger research project with a randomised controlled trial (RCT) design, where an intervention group providing RC efforts is compared to a control group receiving treatment as usual (TAU). The RCT was carried out in the psychiatric care in a large city in eastern Sweden and has been described in a previously published study protocol [23].

### Study setting

The study was conducted at two outpatient departments (hereinafter referred to as care units) that provide care for patients aged 25–64 years with moderate-to-severe-affective and/or anxiety disorders. The HCPs included nurses, psychiatric aides, physicians, psychologists and social workers working at the two care units. Briefly, the primary responsibilities of the various HCPs were as follows: Physician– investigation, assessment, pharmaceutical treatment and issuing sick leave certification; Psychologist– investigation, assessment, psychological treatment and suicide prevention; Social worker– support with housing, financial issues, municipal services, employment and suicide prevention; Psychiatric aide– drug screening, suicide prevention and crisis support; Specialist nurse– pharmaceutical follow-up, suicide prevention, crisis support and coordination with other HCPs. Assessment of suicide risk is conducted by all HCPs.

The RC worked full-time on the assignment, which included the following tasks: assessing patients’ needs for rehabilitation, relevant to their ability to RTW/entry into work or studies; mapping out patients’ rehabilitation needs; designing a rehabilitation plan for RTW/entry into work or studies; initiating and coordinating contacts within healthcare and with external organisations, such as the SIA, PES, municipal services (which, in turn, can initiate efforts or provide support), and employers; acting as a contact person for patients and offering individually tailored support throughout the rehabilitation process. The RC’s assignment also included providing advice and sharing knowledge with HCPs in the units regarding insurance medicine. The RC could also act as an advisor to physicians when writing sick leave certificates.

### Data collection and participants

Data were collected between June 2019 and October 2021 through semi-structured interviews with HCPs. All HCPs in the two care units were invited to take part in the interview study. The department head of the respective care unit delivered an information letter and/or an e-mail provided with information about the aim and procedures of the study. Reminders were sent by e-mail to ensure a variation in the sample.

The HCPs had to contact the researchers themselves to indicate their interest in participating in an interview. The recruitment continued until the authors determined that no new themes were emerging during the interviews.

In total, 15 interviews were conducted, one by the first author (Å.A.) and the rest by the third author (M.C.), who has training and experience in conducting qualitative interviews. The interviews occurred at the care units or by phone, lasting from 23 to 47 min. They were audio-recorded and transcribed verbatim. Two of the interviews were excluded from the analysis due to lack of knowledge about and experience with the RC function. In addition, one interview was excluded due to poor sound quality, as it was not possible to hear what was being said on the recording. Thus, 12 interviews were included, representing the following healthcare professions: physicians– one female and two males; psychiatric aide– one female; nurses– two females; psychologists– two females‚ and social workers– four females.

The researcher used a semi-structured interview guide (see Supplementary Table 1) during the interviews, based on the HCPs’ perceptions of their own role when working with patients on sick leave, their own role in supporting RTW/entry into work, what it meant for them to have a RC at the care unit, and what RCs can contribute to the patients. The last question is reported in another study [11]. The present study focuses on HCPs’ perspectives on what it means for them to have a RC at the care units.

### Methods of analysis

Inductive thematic analysis, as described by Braun and Clarke [24], was used to organise, identify, analyse and report themes within the data, thus answering the research question without following any theoretical framework. Thematic analysis was deemed to be a suitable method for the analysis, as the interviews contained rich and detailed information, thus making it possible to identify patterns in the data [24]. The analysis used a semantic approach and was carried out in six steps, as shown in Table 1

The analysis process was interactive and went back and forth with several revisions (see Supplementary Table 1 for an example of the data analysis process). The analyses were mainly performed by the first (Å.A.) and second (B.C.) authors. The first step was carried out independently. Discussions concerning the coding and themes were held with the last (K.L.) and third (M.C.) authors during the analysis process to reach coherency. Preliminary findings were presented to and discussed with K.L. and M.C., and adjustments were made based on their input. The last author (K.L.) peer-reviewed the analysis during the analysis process. The results were discussed among all authors until a consensus was reached about the final structure. All interviews and the coding were conducted in Swedish. Data extracts and quotes were translated into English by a language editor.

**Table 1:** Steps in the analysis process of data exploring healthcare professionals’ experiences of having access to a rehabilitation coordinator within psychiatric care

The six steps included in the analysis process	
1	The interviews were listened to and the transcriptions were read through to familiarise ourselves with the data. Interview text that addressed the research question, which were judged to be of importance were marked as extracts. Ideas about codes were noted.
2	The marked data extracts were discussed, and data extracts that were assessed as should be included based on their contents were organised into meaningful groups, using preliminary codes. The NVIVO 1.5.1 software programme was used to organise the data.
3	A formal coding of the organised data extracts was performed manually.
4	The different codes were sorted into preliminary themes and sub-themes. Themes and sub-themes were revised several times and checked against each other and back against the original dataset.
5	The coded data extracts were then combined into suggested themes and sub-themes, checking these in relation to each other, leading to further sorting and refining, and to defining and naming the codes, themes, and sub-themes.
6	Results were written.

### The research team

The authors represent one male and five female researchers with different professional backgrounds and expertise: The first author has a PhD in medicine and is a nurse and a public health scientist (Å.A.); the second author is a psychiatric nurse and research assistant (B.C.). The third author is an associate professor and an oncology nurse (M.C.). The fourth author has a PhD in medicine and is an agronomist and a public health scientist (E.B.). The fifth author is an associate professor and behavioural scientist (I.A.). The last author is an associated professor and social worker (K.L.). The first (Å.A), third (M.C.) and last (K.L.) authors have knowledge and experiences in qualitative methods. None of the researchers are clinicians in vocational rehabilitation services. Å.A., E.B., I.A. and K.L. have experience in research within the field of vocational rehabilitation and stakeholder collaboration. While the first (Å.A.), fourth (E.B.) and fifth (I.A.) authors were involved in the planning and evaluation of the RCT, the second author (B.C.) was partially involved in the recruitment of study participants. However, the third (M.C.) and last authors (K.L.) had no part in the planning or execution of the RCT.

## Results

The findings from the interviews are presented based on the two themes: (1) *“A specific function that improves the ability to focus on and support vocational rehabilitation”* and (2) *“Uncertainty and confusion regarding roles and contributions within vocational rehabilitation”*. These themes, in turn, comprise five sub-themes. The themes and their corresponding sub-themes are presented in the text with illustrative quotes. Table 2 provides an overview of the themes and sub-themes, illustrating the healthcare professionals’ experiences of having access to a rehabilitation coordinator in their work.

**Table 2:** Two themes and five sub-themes illustrate the healthcare professionals’ experiences of having access to a rehabilitation coordinator within psychiatric care

Themes	Sub-themes
A specific function that improves the ability to focus on and support vocational rehabilitation	Advantages for the working situation of having access to a rehabilitation coordinator
	Value and use of the rehabilitation coordinator’s knowledge and coordinating role
	Collaboration with a rehabilitation coordinator in the work with patients
Uncertainty and confusion regarding roles and contributions within vocational rehabilitation	Doubts and worries about the rehabilitation coordinators’ assignments
	Lack of clarity in roles and work content

### Theme 1: A specific function that improves the ability to focus on and support vocational rehabilitation

This theme encompasses various aspects of having access to a RC within the care units. The HCPs were generally positive about the introduction of the RC role, viewing it as an improvement in their working situation. The RC’s specific responsibilities were seen as enhancing the ability to offer patients coordinated care and VR. This theme includes the sub-themes: “Advantages for the working situation of having access to a rehabilitation coordinator”, “Value and use of the rehabilitation coordinator’s knowledge and coordinating role” and “Collaboration with a rehabilitation coordinator in the work with patients”.

#### Advantages for the working situation of having access to a rehabilitation coordinator

The HCPs highlighted the RC role as a new function within psychiatry, viewing it as a tool that could increase the prerequisites for the care units to work with VR. They explained that, before the introduction of the RC, they had often observed patients needing coordination and support for VR efforts but were unable to meet their needs due to time constraints and/or lack of knowledge, which led to feelings of inadequacy.

No, but it’s just that this is probably something that has been sorely missed anyway, because we have so many patients who may have never been employed or who have been on sick leave many times or for long periods and we haven’t had it…maybe not always the knowledge or the focus and the opportunities to give the patients what they actually need. (Nurse 1)

The introduction of the RC within the care units was also described as contributing to more extensive VR efforts than what other HCPs with broader working responsibilities could manage. This was also viewed as contributing to increasing attention on the topic within the units and reducing the risk of VR efforts being down prioritised in favour of other work tasks, as one HCP expressed: *Someone who doesn’t then get overwhelmed with suicide prevention or supportive conversations or anything like that*,* but who can just focus on that [VR]. I think that is a rather important function.* (Psychologist 1)

A stated function with specific responsibilities and allocated time to work on VR, i.e. coordination, was considered as creating good conditions for the RC to acquire knowledge and to stay up-to-date with insurance medicine and different efforts related to VR. This also meant increased possibilities to offer rehabilitation coordination as well as follow-up on the VR process. Consequently, the efforts undertaken by RCs were described as valuable, and it felt good for the HCPs to know that the patients could receive this type of dedicated support, as one HCP stated: *So*,* I think it is very nice for the care unit to be able to offer that effort*,* because it is so important.* (Nurse 2). The RC’s work was perceived as positive as it gave the HCPs the possibility to focus on their own main assignment, which also helped to reduce their workload and stress.

But I think, I see it as something positive and helpful, above all for the patients but also for us as staff because it contributes to some form of diffuse stress that, like OK, that’s a very important task that I don’t really have time for. That’s how you experience it most of the time. (Physician 2)

#### Value and use of the rehabilitation coordinator’s knowledge and coordinating role

The RC was described as a valuable resource within the care units, providing specific and in-depth knowledge in the field of VR. This included current research, laws and regulations; the function of the involved authorities and their responsibilities; and how the VR process works. Several HCPs expressed that they had limited knowledge and education regarding VR and also limited time to search for this information on their own. Thus, they found working with VR to be challenging. Having access to a RC meant, for example, that HCPs did not have to spend time keeping themselves updated in the field. Moreover, they could get help or delegate complicated questions from the SIA to the RC, as one HCP expressed: *… you can consult someone*,* knowing that the RC is on site*,* that there is someone who can help with top expertise in more complex cases…* (Physician 1).

… there becomes one more person (RC) there who has some sort of other knowledge whom you can discuss with and precisely because there are many (patients) here who are on long-term sick leave or they will be on sick leave… go back to work for a very short time and then they are on sick leave again, that then… you need someone who has a little more time to get involved and motivate and help out… (Physician 3)

The fact that the RC took responsibility for coordinating patients’ RTW/entry into the work process was viewed as a relief for the HCPs, who found such tasks to be challenging, time consuming and stressful, often taking time away from other duties. However, even though working with VR was deemed important, it was sometimes done in a half-hearted manner.

… I’d think it was very nice because then I’d kind of not have to do this part, actually… to be completely honest. And plus, so… it feels like I’m doing it a bit with my left hand now, like that. (Social worker 1)

The RC’s ability to establish and maintain contact with actors outside the healthcare service, i.e. involved stakeholders, was seen as facilitating the HCPs’ work, as these contacts were sometimes perceived as difficult to initiate and maintain. Moreover, the HCPs felt a sense of reassurance, knowing that patients were being supported by the RC.

So, it’s great. Because I feel, sometimes I call the Social Insurance Agency or Public Employment Service, and the problem is that I don’t really speak their language, I think. I don’t know what all this stuff is, which means that I can sometimes have a hard time understanding or knowing what is going on. (Psychologist 2)

And that’s really great, because I think that you really need special knowledge about who you contact, how you sort of do it, who needs to be involved. It feels like it’s a huge task to understand how the Swedish Social Insurance Agency works, how it is with different efforts that exist, and so on. So it’s great that someone has a firm grasp on it; it feels like a chapter of its own. (Psychologist 1)

By sharing information about available VR efforts, the RC was seen as increasing the HCPs’ understanding of these processes. In addition, the RC was viewed as raising awareness among HCPs about the patients’ specific VR needs. The RCs actions helped the HCPs to feel relieved, as well as reduced stress and frustration, as these tasks meant extra work for them and could, for example, spare them from feelings of guilt for not having enough time to do these tasks themselves.

I don’t know what these efforts are; now I have gradually learned what is usually talked about at these coordination meetings and in the contact with RC, so I have also been able to gain a little better understanding of what these efforts entail. (Physician 1)

We, social workers, said that RC is incredibly important. I would really like more. Especially in specialist psychiatry here, I would like more people to do what the RC does. Helping people into employment and just being able to be there and actually also have time to find out what is applicable, what are the new rules, what are the new laws, exceptions, in other words, a lot of things that we social workers who have other forms of conversation, have to deal with after regular working hours actually because we don’t have time during the day. (Social worker 3)

#### Collaboration with a rehabilitation coordinator in the work with patients

HCPs found it valuable to collaborate with the RC, as it allowed them to make use of the competence of the different HCPs in the work with patients. Collaboration with the RC also enabled a common planning for the work according to the patients’ VR processes. It also made it possible to distribute different work tasks among each other. This was seen to facilitate the HCPs’ work with the patients.

Then we have been able to collaborate on making a small list of things like this that need to be worked on. I can share that list with the RC as well, which the RC can also follow-up on, like, where are we and where, I know that the patient can also work with it. So, it becomes a good collaboration where you can share a little and plan together. That makes it easier for me, too. (Psychologist 1)

The collaboration provided valuable and specific information to the HCPs about the patients, based on the RC’s mapping of, and work with, the patients. This information could be used by other HCPs in their assessments of the patient and could therefore contribute to an increased understanding of the patient’s needs. Through the work of the RC, the HCPs were given the opportunity to follow and receive feedback regarding the VR process and thereby gain a better overview of how this process was progressing for the patient.

… then I know that the patient will be well taken care of. I know that I will get feedback if problems arise; I know that I will be told if the patient should cancel or stop. I know what happens in a different way than if the patient does not have that kind of contact. So, I know what happens with the rehabilitation but also that it feels good… (Nurse 1)

The HCPs also observed that the information the RC shared about the patients facilitated and saved time for them in their own work with the patients. In addition, the RC could guide other HCPs on how they could contribute to the VR process, which was not always clear. This was viewed as both increasing the quality of the VR process and being time efficient. Moreover, collaboration with the RC enabled shared responsibilities and information about patients, thus facilitating the HCPs’ work and was perceived as improving the quality of the VR process.

And it’s great for us here who are running around and kind of don’t have a clue, so that we… when the RC sends out a template and stuff like this; this is what you need to find out for this time or something like that, then it is always clear; it is very effective… (Social worker 3)

### Theme 2: uncertainty and confusion regarding roles and contributions within vocational rehabilitation

The introduction of the RC also raised some concerns and ambiguities regarding the RC function and its role in VR. These uncertainties, to some extent, also included the HCPs’ own work, with some expressing reservations about the need for validation and a desire to see results regarding the benefits of the RC function. There was also a fear that introducing RCs would mean changes in staffing within the care units. This theme included the sub-themes: *“Doubts and worries about the rehabilitation coordinators’ assignments”* and *“Lack of clarity in roles and work content”.*

#### Doubts and worries about the rehabilitation coordinators’ assignments

Despite a generally positive perception of the introduction of RCs, there was some uncertainty about their function, with suggestions for evaluating it with respect to benefits and costs. HCPs wanted to know if the RCs’ work was helpful for the patients before they were convinced about their usefulness. There were also concerns that introducing RCs into care units would, in the long term, lead to a reduction in other HCPs’ roles.

… I mean, a certain fear can be felt when you ask that question, as if there is some kind of crowding out effect from, for example, nurses, as if; yes, we have this bag of money and then we take some away from the nurses and hire a RC person. (Physician 2)

Different opinions were expressed about whether, and if so, where VR should be performed within the healthcare system. For example, some believed that VR (and thus the placement of RCs) would be better suited within the framework of primary care, while others felt that RCs play an important role within psychiatric care.

… this is not really the main task of healthcare services, to get people into work or to get people to start studying and so on, but it is clear that it is a very important part of recovery, but it is not our primary task. (Social worker 1)

The objection here would be that, again, perhaps rehabilitation should be something that is handled at primary care level, in the best of all worlds. (Physician 1)

#### Lack of clarity in roles and work content

There was some uncertainty about how the RC operated. For example, HCPs assumed that part of the RCs’ work involved finding solutions and opportunities for employment tailored to the patient’s conditions. However, their knowledge about the RC’s work in practice varied.

I believe it’s a very important function (rehabilitation coordinator), but I’m not very familiar with the specific details of what they do. (Nurse 2)

.. I don’t know too much about how RC works, but I think the RC has been a positive force for the patients I have met. Then, I don’t know exactly what those things look like. (Psychologist 2)

Since the RC’s tasks were, to some extent, similar to those of the social workers, HCPs described the differences between these roles as unclear. However, it was emphasised that both functions were needed. The RC’s role was seen as complementary to that of social workers, though the social workers’ responsibilities were broader, with support for employment being just one of several duties.

But I can really see, I don’t think it excludes one or the other. Like, I think RC is a very important professional role; I think here at the care unit, there would be tasks that were not done if the RC was not here, I think. (Social worker 3)

There were also uncertainties among HCPs about their own roles in VR, and for some, it was unclear whether, and if so, how they should contribute to supporting patients’ entry into employment within the scope of their own professional responsibilities. Sometimes, they found themselves handling work tasks related to sick leave or VR, even though these were considered outside their regular duties, as one HCP described:

… because it’s not really something I have to work with, but it’s something that all the time… pops up, like if there is a problem with the Social Insurance Agency that affects the treatment. (Psychologist 2)

## Discussion

The aim of this interview study was to explore HCPs’ views and experiences of having access to a RC in their work within psychiatric care. The analysis identified two key themes, illustrating that HCPs generally viewed the introduction of the RC function within care units positively. The RC role was seen to enhance the ability to focus on and provide patients with coordination and VR efforts, while also alleviating HCPs’ own workload. However, some uncertainty and confusion were reported regarding the RC’s function and role, as well as the HCPs’ responsibility in relation to VR.

The HCPs perceived the RC as a valuable complementary resource within the psychiatric healthcare units. The RC’s specific focus and well-defined assignment were seen as contributing to more comprehensive efforts to support patients throughout the VR process. HCPs found collaboration with the RC beneficial, as it provided them with information and feedback about patients’ VR progress and guidance on how to best support them. This collaboration also enhanced general knowledge in the VR field and allowed for better use of the diverse expertise of different professionals involved in patient care. The RCs’ work was thus perceived to reduce HCPs’ workload and alleviate work-related stress. Similar findings were reported in a qualitative evaluation involving physicians in primary care, who expressed that RCs helped to ease their workload [25].

The RCs’ responsibility for coordination was viewed as positive and helpful, providing HCPs with a sense of assurance when the RC took responsibility for these tasks. This aligns with findings from other studies involving physicians (GPS and psychiatrists), which highlight that contact with external VR actors is often experienced as demanding [26, 27] and is facilitated by the involvement of RCs [27]. It is worth noting that the primary VR actor mentioned by the HCPs was the SIA. This may be natural given that the SIA makes decisions about patients’ entitlement to sick leave benefits. As the VR process often involves numerous actors and professions, as shown in a Norwegian study by Skumsnes et al. [28], RCs may function as dedicated professionals who maintain an overview of relevant information and ensure overall coordination. The involvement of several actors, each with different areas of responsibility in the VR process [30], entails that collaboration between them is crucial for achieving a positive outcome [31]. Besides the number of actors, other factors such as the patient’s social life situation, health condition, attitudes, work experiences and work-related factors all influence the patient’s workability, adding complexity to the VR and RTW/entry into work process [29]. This requires specialised knowledge, which the HCPs in our study felt they did not possess.

Before the introduction of the RCs, no HCPs within the Swedish healthcare system had formal responsibility for supporting patients through the sick leave and VR process, or for collaborating with external actors [30]. Although support for employment was available, e.g. via social workers, this was only one aspect of their broader role. Even though HCPs in our study recognised the importance of supporting patients in RTW/entry into work or studies, and acknowledged the need for such support within the target patient group, they usually did not have enough time for such efforts. Additionally, several HCPs felt that their knowledge of VR and insurance medicine was limited, with insufficient time to research and update this information themselves. Similar results have been reported in a previous study, where psychiatrists described a perceived lack of competence regarding e.g. insurance medicine and VR methods, as well as difficulties in understanding and gaining an overview of the VR process [26]. Furthermore, general practitioners in primary care, working with patients who have common mental disorders, perceived the RC to be helpful, contributing valuable knowledge and information in the field [27].

Not being able to meet patients’ needs for coordination and support in VR efforts (before the implementation of the RC), led to feelings of inadequacy and frustration among the HCPs, as the working situation was not compatible with their own values. A similar finding has been reported by professionals from different VR agencies [31]. In addition, not being able to offer patients the support they require can lead to moral distress (i.e. feelings that arise when someone knows the morally correct course of action but is unable to take it [32]). Over time, this may have negative effects on the HCPs’ health. One explanation for the lack of focus on VR in ordinary work may be that HCPs need to prioritise more urgent/fundamental tasks, such as medical examination and treatments, as well as a perceived lack of knowledge in the area.

Healthcare services should contribute to supporting patients’ RTW/entry into work and are responsible for assessing patients’ needs for sick leave, treatment, rehabilitation and coordination efforts. They should also collaborate with other actors if needed. The specific investigations and treatments required by the patient determine which HCPs need to be involved [33]. However, not all HCPs in our study considered it to be a primary task of healthcare services to manage the patient’s VR. Some thought that VR should mainly be managed within primary care. Furthermore, according to their own professional assignment, some HCPs expressed uncertainty about their own responsibilities in the patient’s VR process. This can lead to stress for HCPs and result in variations in commitment and work across different HCPs involved in supporting the patient’s VR process. This lack of clarity regarding the roles, responsibilities and values of different professionals can influence actions during the VR process, which entails potential consequences for the patients, including the risk of unequal care [34].

We also found varying degrees of uncertainty among the HCPs about how the RCs worked. Implementation research stresses that a lack of clarity regarding roles and responsibilities can act as a barrier to the successful implementation of new methods [35]. This shows the importance of clarifying both the roles of the HCPs and the roles of RCs in order to improve HCPs’ support in the VR process, optimise the use of RCs and facilitate the implementation of RCs within psychiatric care.

According to Swedish law, the coordination service for patients on sick leave assigns responsibility for defining the content and scope of the RCs’ work to the healthcare regions and local managers. As a result, the RCs’ assignment and their extent, training and tasks vary between healthcare services. This places great responsibility on managers to clearly define the function and role of RCs within their specific healthcare units. For the successful implementation of RCs, managers emphasise the importance of providing RCs with support and the mandate necessary to carry out their mission effectively within the healthcare unit [36].

## Methodological considerations

To ensure the trustworthiness of the data, several strategies were used [37]. To enhance credibility, both male and female HCPs representing various professions were invited to participate in the study, ensuring a variation in perceptions and perspectives on the research question. Credibility was further ensured by presenting the findings together with citations.

Dependability [37] was enhanced by using the same question areas for all HCPs during the interviews. Moreover, dependability was achieved by being transparent with the description of the research process, making it possible to follow the research process and replicate the study. The authors’ different backgrounds and experiences in vocational rehabilitation research contributed to pre-understanding within the research field, which could be seen as a strength. As two authors had no prior background in the research area, the risk of being blinded to new perspectives and insights was minimised. The results were discussed among all authors until a consensus was reached on the final structure (for examples of the analysis process, from data extraction to themes, see supplementary, Table 1).

Transferability [37] was achieved through a detailed description of the context, the selection and recruitment of the study participants (HCPs), their professions and genders, as well as the processes for data collection and analysis. However, the transferability of the findings in this study may be limited due to variations in the RC’s practices, both within Sweden and across countries. In addition, the health insurance systems and actors involved in the VR process may also differ across countries. Despite these limitations, the results may be valuable and of interest to health organisations and actors involved in vocational rehabilitation.

The study has several limitations that need to be considered. Data collection took place over a long time-period, mainly because of the COVID-19 pandemic, during which time the interviews had to be paused. Before the COVID-19 pandemic, the interviews were conducted face-to-face; however, after the pause, they had to be carried out by telephone, leading to a variation in the data collection method. Nonetheless, no changes were evident in the quality of the interviews due to this change. The second reason why the data collection took time was connected to the expansion of the intervention to a second care unit in the spring of 2021. This may pose a risk of inconsistency in the data. Furthermore, a limitation concerns the HCPs’ experiences and knowledge of the RCs’ work, which varied among the HCPs. The reason for this was partly due to the later introduction of the intervention at one of the care units included in the study. Some HCPs also shared their thoughts on what the RCs could contribute to their work. There were also two HCPs interviewed who lacked knowledge about the RCs’ function and their work at the relevant care units; consequently, these interviews were excluded from the analysis.

## Conclusions and clinical implications

The efforts of the RC were perceived positively and considered valuable, as they increased the focus on VR, which is a complex and time-consuming process, and extended support to patients throughout the VR process. Although HCPs recognised the importance of VR, they felt they lacked both the time and knowledge to manage these challenging tasks within their current work situation. Consequently, the RC function filled a gap within psychiatric healthcare units and contributed to an improved work environment for HCPs, potentially reducing work-related stress. However, it is essential to clarify the various roles and responsibilities involved in the VR process to enhance the use of RCs and minimise inequalities in patient care. Moreover, insufficient focus on VR may lead to fewer individuals with mental disorders RTW/entering work or studies, which could have negative consequences for both the individual and society. This study highlights the gap that RC function fills from the perspective of HCPs, addressing their experiences with aspects of both their own role and the RC’s role within the VR process that they found unclear. As a result, this study’s findings may help guide and support the implementation of RCs across various care units, potentially extending beyond psychiatric care.

## Supplementary Information

Below is the link to the electronic supplementary material.

Supplementary Material 1

Supplementary Material 2

## Data Availability

The data presented in this study are not available due to ethical restrictions.

## Abbreviations

RC: Rehabilitation coordinator
HCP: Health care professional
RTW: Return to work
VR: Vocational rehabilitation
SIA: The social insurance agency
PES: The public employment service
RTWC: Return to work coordinators
