# Supplementary material for: Exploring healthcare professionals’ views and experiences of having access to a rehabilitation coordinator in their work in psychiatric care: a qualitative descriptive study
Source: BMC Health Serv Res. 2026 Oct 27;26:1478. doi: 10.1186/s12913-026-15919-8 (PMC13715159; doi:10.1186/s12913-026-15919-8)
Supplement: Supplementary file 1 [file 12913_2026_15919_MOESM1_ESM.docx]

**Interview guide**

How have HCPs experienced the availability of a rehabilitation coordinator within the healthcare unit?

- Possible advantages/disadvantages?
- Areas of improvement?

1. How do you perceive your role in working with patients on sick leave?
2. How do you view your professional role in supporting patients’ transition to employment?
3. What does having a rehabilitation coordinator at the healthcare unit mean to you as a HCP?
4. What do you think a rehabilitation coordinator can contribute to the patients?
5. Is there anything you would like to share that hasn’t been asked?
