# Supplementary material for: Exploring healthcare professionals’ views and experiences of having access to a rehabilitation coordinator in their work in psychiatric care: a qualitative descriptive study
Source: BMC Health Serv Res. 2026 Oct 27;26:1478. doi: 10.1186/s12913-026-15919-8 (PMC13715159; doi:10.1186/s12913-026-15919-8)
Supplement: Supplementary file 2 [file 12913_2026_15919_MOESM2_ESM.docx]

**Table 1.** Examples of data analysis from data extract to themes.

| **Data extract** | **Codes** | **Sub-themes** | **Theme** |
| --- | --- | --- | --- |
| That the rehabilitation coordinator sort of... specific and focuses on that part and I think... absolutely, a rehabilitation coordinator makes a difference; it's also good for us (the HCPs) if the RC does that part.  (Social worker 2) | Difference for other professions if the rehabilitation coordinator can focus specifically on vocational rehabilitation | Advantages for the working situation of having access to a rehabilitation coordinator | A specific function that improves the ability to focus on and support vocational rehabilitation |
| So, I am very positive about the rehabilitation coordinator under the assumption that these efforts actually help the patients…  (Physician 1) | Positive towards having a rehabilitation coordinator provided that it promotes work | Doubts and worries about the rehabilitation coordinator’s assignments | Uncertainty and confusion regarding roles and contributions within vocational rehabilitation |
